# Premature Ovarian Insufficiency (POI) Induced by Dynamic Intensity Modulated Radiation Therapy via P13K-AKT-FOXO3a in Rat Models

**DOI:** 10.1155/2021/7273846

**Published:** 2021-06-29

**Authors:** Lianli He, Xiaoqin Long, Nixiao Yu, Yajun Li, Xiaoyun Liu, Xiaoju Cheng

**Affiliations:** ^1^Department of Gynecology and Obstetrics, The First People's Hospital of Zunyi and Third Affiliated Hospital of Zunyi Medical University, Zunyi, 563000 Guizhou, China; ^2^Oncology Department, The First People's Hospital of Zunyi and Third Affiliated Hospital of Zunyi Medical University, Zunyi, 563000 Guizhou, China; ^3^Central Laboratory, The First People's Hospital of Zunyi and Third Affiliated Hospital of Zunyi Medical University, Zunyi, 563000 Guizhou, China

## Abstract

This study is aimed to investigate the mechanisms of radiation-induced mouse models of premature ovarian insufficiency (POI). Wistar female rats were grouped into the control, 3.2 Gy, 4.0 Gy, and 4.8 Gy groups. Overall ovarian functions were assessed with the H&E staining and ELISA. Proinflammatory cytokine secretion was analyzed ELISA, and the reactive oxygen species (ROS) levels were analyzed with immunohistochemistry. Protein expressions were analyzed by Western blot analysis. The 4.0 Gy and 4.8 Gy groups had significantly lower ovarian weight coefficients than the control and 3.2 Gy groups (after only one irradiation therapy). The 3.2 Gy radiation group induced periodic disturbance and hormone change at 4 weeks after radiation. In the 4.0 Gy and 4.8 Gy groups, the preantral follicles and antral follicles were decreased, while Atresia follicles were increased. E2 was decreased, while FSH and LH secretions were increased. The ovaries in the 4.0 Gy group were not completely atrophied, and some preantral follicles remained. Ovarian atrophy and follicular Atresia were found in the 4.8 Gy group. Inflammatory and oxidative markers were upregulated. PI3K and AKT were downregulated in the 4.0 Gy and 4.8 Gy groups, while FOXO3a was upregulated. Ovarian injuries may lead to oxidative damages and inflammatory injuries, downregulate the expression of P13k and Akt, upregulate the expression of FOXO3a, and lead to follicular atresia in the ovary.

## 1. Introduction

The ovary is a female sex gland, which has the function of producing mature egg cells and the endocrine function of producing female hormones. It plays an important role in maintaining female menstruation, reproductive physiology, and fertility. The development process of mammalian follicles in the ovary mainly includes primordial follicles, preantral follicles, antral follicles, and preovulatory follicles. The ovary is highly sensitive to radiation, and primordial follicles appear to be the most sensitive among all stages of follicles [1]. Generally, the major mechanisms of ovarian injuries include follicle cell apoptosis, oxidative stress, ovarian atrophy, cortical fibrosis, and blood-vessel damage [2–4]. However, radiation causes damage mostly via ionization and formation of reactive oxygen species (ROS), and the increased ROS induces rapid primordial follicle loss in the ovaries [5, 6].

Premature ovarian insufficiency (POI) is a hypergonadotropic ovarian deficiency featured with elevated gonadotropin and low estrogen levels, affecting approximately 1% of women before the age of 40 years old [7]. The underlying etiology for POI is complex and remains to be revealed. Radiation therapy, which has well-documented toxic effects on the reproductive system and the fertility of women, is one of the important etiologies for POI [8, 9]. The hallmarks of radiation-induced POI are menstrual disorder, infertility, osteoporosis, psychological problems, and impaired quality of life [7].

POI is irreversible, but early detection of ovarian failure allowing for a timely diagnosis in conjunction with early treatment can possibly delay or even improve the condition. Therefore, identifying the underlying mechanism of POI is critical for the treatment of this disease. In this study, rats were treated with different doses to explore the best dose for the model establishment, and the expression of the inflammatory index, oxidative index, and P13K-AKT-FOXO3a signal pathway was detected. Our findings may lay a solid foundation for our follow-up research on treatment options.

## 2. Materials and Methods

### 2.1. Study Animals and Model Establishment

A total of 24 Wistar female rats with normal estrus, 10-11-week old, were purchased from the Liaoning Changsheng Biotechnology Co., Ltd. Rats were fed adaptively for one week before experiments in a standard condition, with free access to food and water. All animal procedures were approved by the Ethics Committee of the First People's Hospital of Zunyi and Third Affiliated Hospital of Zunyi Medical University (approval no. 2018-043).

**Figure 1:**
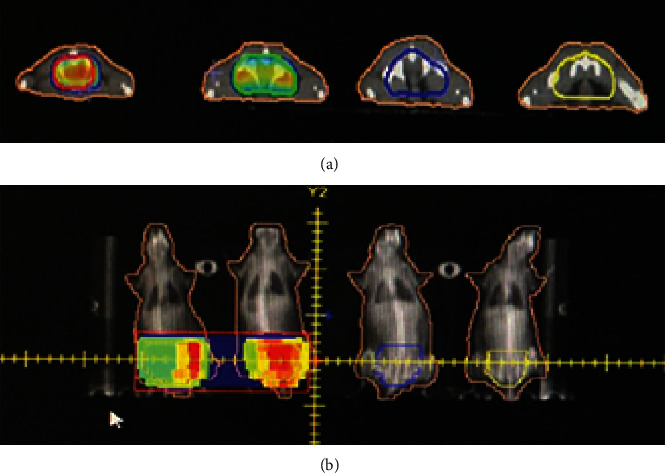
Pelvic target area and irradiation area. The pelvic target area (a) and the irradiation area (b) were delineated.

The radiation dosage in this study was calculated according to the maximum and minimum radiation doses for gynecological tumor patients based on the body surface area. The rats were divided into the following groups (*n* = 6): the control, 3.2 Gy, 4.0 Gy, and 4.8 Gy groups. For the radiation groups, the rats were intraperitoneally injected with 10% glutaraldehyde in the Tumor Radiotherapy Center of our hospital. Under CT localization, the pelvic irradiation target area was located and delineated by the technicians (avoiding heart, liver, spleen, lung, and kidney) using the precise VMAT of a medical linear accelerator, with the irradiation distance of 1 meter (Figure 1). The single irradiation dosages were set as 3.2 Gy, 4.0 Gy, and 4.8 Gy, respectively. For the control group, the rats were anesthetized with glutaraldehyde, with no radiotherapy. The rats were observed every two weeks for 8 weeks after irradiation.

The vaginal smears were obtained between 8 : 00 AM and 9 : 00 AM every day, which were analyzed every two weeks to determine the changes in the estrous cycle. The blood sample was obtained from the tail vein every 2 weeks. When the vaginal exfoliated cells were collected and the hormone levels were detected, the POI models would be killed after anesthesia with 10% glutaraldehyde. Moreover, the weights of the ovaries and the uterus were recorded. All animal experimental procedures were in accordance with the National Research Council Guide for Care and Use of Laboratory Animals.

### 2.2. ELISA

At 2 weeks after radiation, the levels of Mouse Estradiol 2 (E2), follicle-stimulating hormone (FSH), and luteotropic hormone (LH) in the blood samples were detected. After incubation at room temperature for 1 h, the serum was collected through centrifugation at 5000 rpm for 10 min. The sex hormone levels were determined by the ELISA kits (Taisiteng Biotechnology Co., Ltd., Guiyang, Guizhou, China), following the manufacturer's instructions. Moreover, for the detection of proinflammatory cytokines, the ovarian supernatant was collected, and the levels of interleukin-1*β* (IL-1*β*), IL-6, and tumor necrosis factor-*α* (TNF-*α*) in the ovaries were detected by the ELISA kits (Taisiteng Biotechnology Co., Ltd.), following the manufacturer's instructions.

### 2.3. Papanicolaou Staining

To analyze the estrous cycle, the vaginal exfoliated cells in the estrous cycle of rats were subjected to the Papanicolaou staining. Briefly, the vaginal exfoliated cells were collected as previously described [10]. The staining procedure [11] included 6 slow dips under tap water, stained with Harris hematoxylin for 30 s; 6 slow dips under tap water; 6 dips in 95% isopropyl alcohol, stained with EA-36 for 15 s; 6 dips in 95% isopropyl alcohol; 6 dips in 100% isopropyl alcohol; and 10 slow dips in xylene. Finally, the sections were mounted and observed under a microscope. Normal estrous cycles included the diestrus, proestrus, estrus, and metestrus cycle stages. Only rats with full cycle changes were defined as having normal estrous cycles. The percentage of normal cyclicity, which was statistically analyzed every 2 weeks, was defined as the ratio of the number of rats with normal estrous cycles to the total number of rats in each group.

### 2.4. Hematoxylin and Eosin (H&E) Staining

The maturation of ovarian follicles was analyzed by HE staining according to the previous description [12, 13]. After anesthesia, the organs (livers, lungs, spleens, kidneys, and ovaries) were removed from the rats. After fixed with 4% paraformaldehyde at 4°C overnight, the samples were dehydrated, transparentized with xylene, and embedded in paraffin. The embedded organs were cut into 5-*μ*m sections and were stained with H&E. The histological examinations were performed under microscopy. Images were obtained and analyzed with the Image-Pro Plus 6.0 software (Media Cybernetics Inc., Atlanta, GA). The ovarian follicles were detected and classified as preantral follicle, antral follicle, and atretic follicle, according to the previous description [14, 15]. Follicle counts at all levels were performed as previously described [16] by a professional pathologist, in a single-blinded way. Only the follicles that contained a clearly visible nucleus and an oocyte were counted.

### 2.5. Immunohistochemistry

Immunohistochemical analysis was performed to detect the ROS levels, as previously described [17]. Briefly, the ovaries were washed with PBS for three times, which were then fixed with 4% paraformaldehyde for 30 min, dehydrated with graded ethanol, transparentized with xylene, and embedded in paraffin. The samples were cut into 5-*μ*m serial sections, which were subjected to the microwave antigen retrieval, following rinsing with 3% phosphate buffer. After blocking, the sections were incubated with the primary polyclonal antibodies (1 : 100 dilution; Uning Biotech, Shanghai, China) at 4°C overnight. After washing, the sections were incubated with the horseradish peroxidase-conjugated secondary antibody at 37°C for 45 min. The color development was performed with the avidin-biotin complex chromogenic reagent (Beyotime, Shanghai, China). For each tissue section, five fields were randomly selected from each view (×200 magnification). The relative optical density (ROD) values of ROS were analyzed with the Intel Integrated Performance Primitives software Version 6.0 (Media Cybernetics, Rockville, MD, USA).

### 2.6. Western Blot Analysis

Total protein was extracted by the RIPA buffer (Mengbio, Chongqing, China). Protein concentrations were detected by the BCA method (Beyotime). The protein samples were separated by the SDS-PAGE, which was then transferred to the PVDF membrane. After blocking with 5% nonfat milk, the membrane was incubated with the rabbit anti-mouse primary antibodies of anti-P13K, anti-AKT, anti-FOXO3a, and anti-GAPDH (1 : 100 dilution; Uning Biotech) at 4°C overnight. Then, the membrane was incubated with the goat anti-rabbit secondary antibody (1 : 500 dilution; Uning Biotech) at room temperature for 1 h. The color development was performed with the enhanced chemiluminescence (ECL) detection system (Thermofisher, Waltham, MA, USA). The protein bands were imaged and analyzed with the Quantity One software (Bio Rad). GAPDH was used as an internal reference.

### 2.7. Statistical Analysis

Data were presented as mean ± SD. The SPSS 18.0 software was used for data analysis. One-way ANOVA was used to compare the differences in mean values among the groups, with the LSD test for the multiple comparisons. *P* < 0.05 was considered statistically significant.

## 3. Results

### 3.1. Radiation Decreases Ovary Weights

**Figure 2:**
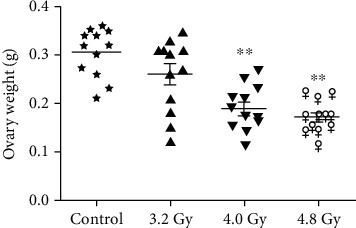
Analysis of the ovary weights. The ovary weights in the control, 3.2 Gy, 4.0 Gy, and 4.8 Gy groups were analyzed and compared. Compared with the control and 3.2 Gy groups, ^∗∗^*P* < 0.05.

To investigate the effects of radiation on the rats, the weights of the ovary were analyzed after radiation. As shown in Figure 2, the ovary weights were decreased in the 4.0 Gy and 4.8 Gy groups, compared with the control and 3.2 Gy groups. Moreover, there was no significant difference in the ovary weight between the 4.0 Gy and 4.8 Gy groups. These results suggest that the radiation at high dosages could decrease the ovary weights.

### 3.2. Radiation Disturbs Estrous Cycle of Rats

**Figure 3:**
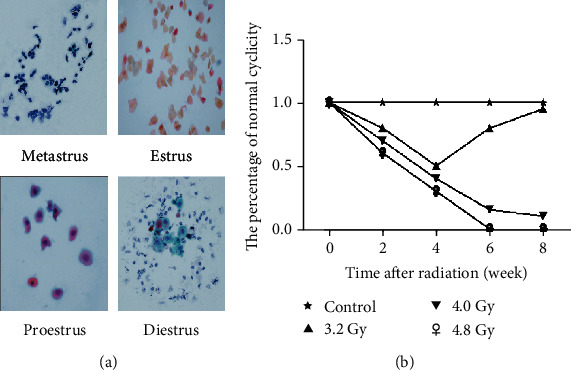
Effects of radiation on the estrous cycle of rats. (a) The vaginal exfoliated cells in the estrous cycle of rats at different irradiation doses were subjected to the Papanicolaou staining (×40). (b) Statistical analysis of the data from the control, 3.2 Gy, 4.0 Gy, and 4.8 Gy groups.

The vaginal smears were stained with Papanicolaou staining to evaluate the estrous cycles. Normal estrous cycles include the diestrus, proestrus, estrus, and metestrus cycle stages (Figure 3(a)). Only rats with full cycle changes were recorded as having normal estrous cycles, and the percentage of normal estrous cycles was statistically analyzed every 2 weeks. Our results showed that the proportions of the rats with normal cycles were decreased for the 3.2 Gy, 4.0 Gy, and 4.8 Gy groups. Moreover, the 3.2 Gy group returned to normal level after decreasing, while the 4.0 Gy and 4.8 Gy groups were decreased most obviously. The 4.8 Gy group had no normal cycle rats from 6 weeks after radiotherapy (Figure 3(b)). These results suggest that the radiation could disturb the estrous cycle of rats.

### 3.3. Radiation Affects Ovarian Follicular Development

**Figure 4:**
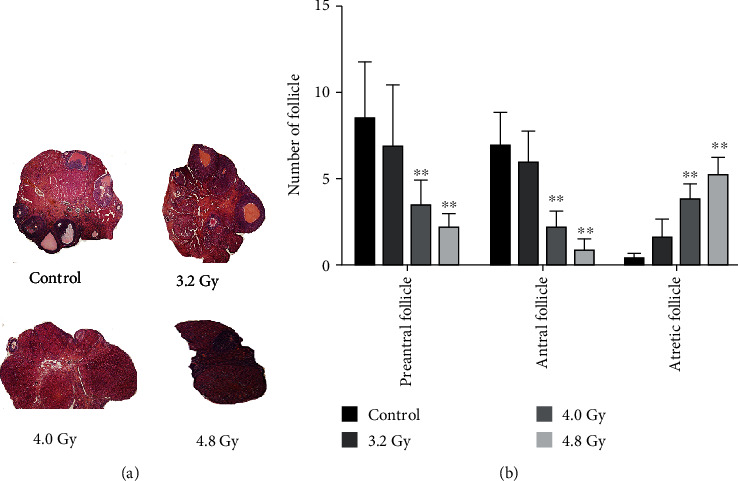
Ovarian H&E staining. (a) The H&E staining was performed to detect the preantral follicles, antral follicles, and atresia follicles in the model rats (×40). (b) Statistical analysis of the data from the control, 3.2 Gy, 4.0 Gy, and 4.8 Gy groups. Compared with the control and 3.2 Gy groups, ^∗∗^*P* < 0.05.

The effects of radiation on the maturation of ovarian follicles were next investigated with the H&E staining. Our results showed that the ovaries of the rats in the control, 3.2 Gy, 4.0 Gy, and 4.8 Gy groups had follicles that were at different stages of maturation after radiation. In contrast, the 4.0 Gy and 4.8 Gy groups exhibited different architecture of the ovaries. Ovaries in the rats from the 4.0 Gy and 4.8 Gy groups had reduced ovarian mass and reduced antral follicles (Figure 4(a)). Moreover, the 4.8 Gy group only had atretic follicles. Results from the ovarian follicle counting showed that the rats from the control and 3.2 Gy group had significantly higher numbers of preantral follicles and antral follicles, compared with the 4.0 Gy and 4.8 Gy groups. There was no significant difference in the number of any kind of follicles between the control and 3.2 Gy groups (Figure 4(b)). The difference between the control group and 4.8 Gy group in follicle number was about 9-10 folds. These results suggest that the radiation could affect the ovarian follicular development.

### 3.4. Radiation Decreases Serum Levels of Gonadotrophins, E2, FSH, and LH

**Figure 5:**
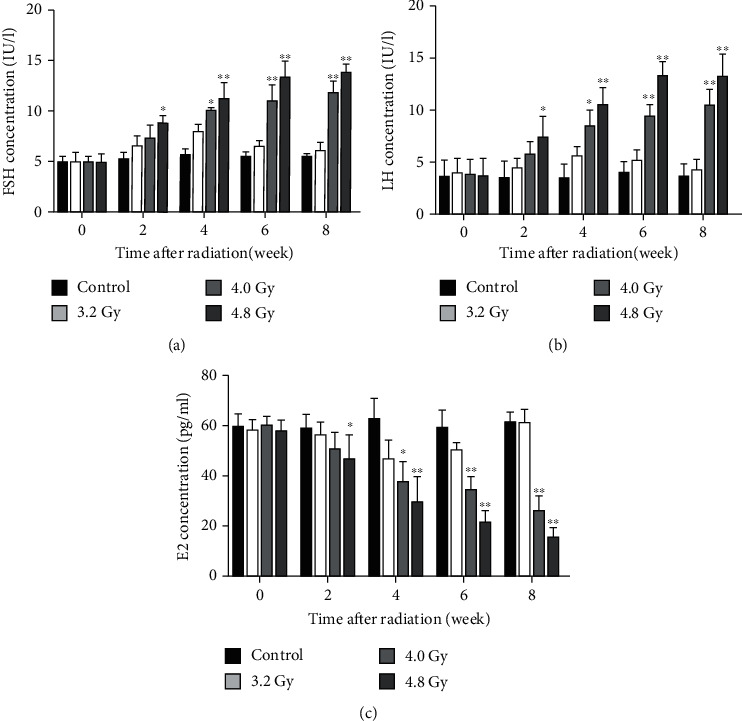
Analysis of FSH, LH, and E2 levels. The levels of FSH (a), LH (b), and E2 (c) in the serum were detected and compared between the control, 3.2 Gy, 4.0 Gy, and 4.8 Gy groups. Compared with the control and 3.2 Gy groups, ^∗∗^*P* < 0.05.

Whether the hormone levels were affected by radiation was then investigated. Our results showed that, as time passed, the E2, FSH, and LH levels in the rats of the control group and 3.2 Gy group had no obvious changes. However, compared with the control group and 3.2 Gy group, along with the time passing, the E2 levels in the rats of the 4.0 Gy and 4.8 Gy groups were gradually decreased, while the FSH and LH levels were gradually increased. Moreover, the most obvious results were observed for the 4.8 Gy group (Figures 5(a)–5(c)). These results suggest that the radiation could decrease the serum levels of gonadotrophins, E2, FSH, and LH in the rat models.

### 3.5. Radiation Increases Proinflammatory Cytokine Levels

**Figure 6:**
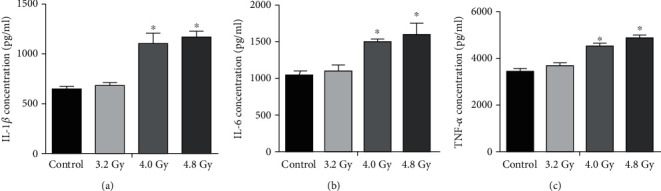
Analysis of IL-1*β*, TNF-*α*, and IL-6 contents. The levels of IL-1*β* (a), TNF-*α* (b), and IL-6 (c) were detected with ELISA, which were compared between the control, 3.2 Gy, 4.0 Gy, and 4.8 Gy groups. Compared with the control and 3.2 Gy groups, ^∗∗^*P* < 0.05.

Increased proinflammatory cytokines, including IL-1*β*, IL-6, and TNF-*α*, have been found in the inflammatory tissues, which are associated with the radiation-induced ovarian injuries. ELISA was then performed to detect levels of IL-1*β*, IL-6, and TNF-*α*. Our results showed that the levels of IL-1*β*, IL-6, and TNF-*α* were significantly increased in the 4.0 Gy and 4.8 Gy groups compared with the control and 3.2 Gy groups (Figure 6). These results suggest that the radiation in the 4.0 Gy and 4.8 Gy groups could increase the inflammation in the ovaries.

### 3.6. Antioxidative Effects of Radiation on Ovaries

**Figure 7:**
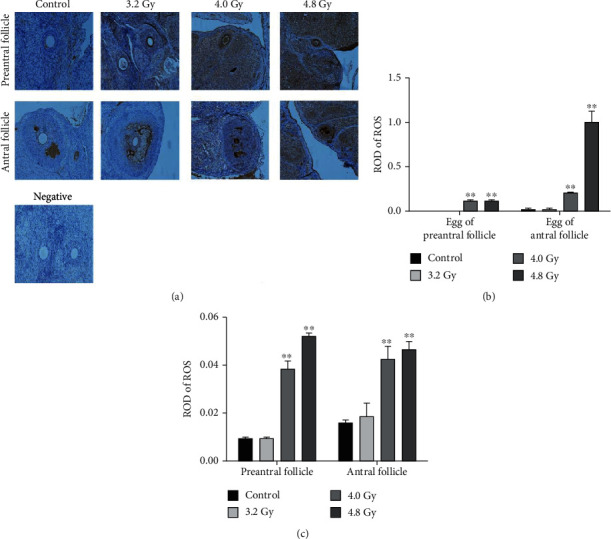
Analysis of the ROS levels in the preantral follicles and antral follicles. (a) The ROS levels in the preantral follicles, antral follicles, and their oocytes were detected with immunohistochemistry. (b) Statistical analysis of ROS level in the egg of preantral follicles and antral follicles from the control, 3.2 Gy, 4.0 Gy, and 4.8 Gy groups. (c) Statistical analysis of ROS level in the preantral follicles and antral follicles from the control, 3.2 Gy, 4.0 Gy, and 4.8 Gy groups. Compared with the control and 3.2 Gy groups, ^∗∗^*P* < 0.05.

To determine the effects of radiation on the cellular localization of ROS within the ovaries, immunohistochemical analysis was performed. Our results showed that, in the granule cells of rat ovaries, the ROS production was observed only in the preantral follicle and antral follicles (Figure 7(a)). The intensities of ROS in the follicles were assessed by ROD values (Figures 7(b) and 7(c)). The ROD for ROS was significantly higher in the 4.0 Gy group compared with the control and 3.2 Gy groups. Moreover, rats in the 4.0 Gy and 4.8 Gy groups had significantly higher ROS levels than the control and 3.2 Gy groups. The results demonstrate that higher dosages of radiation could increase the ROS production in the granule cells of preantral and antral follicles.

### 3.7. Radiation Affects Expression of P13K-AKT-FOXO3a Proteins

**Figure 8:**
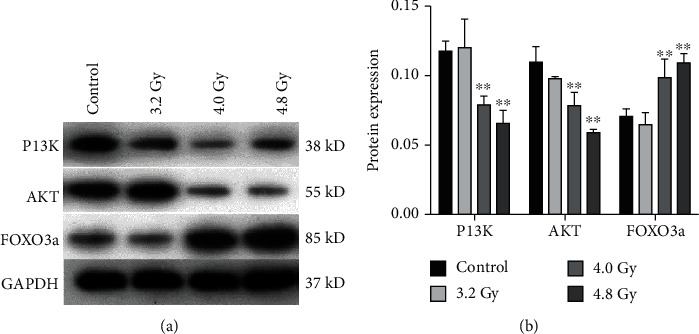
Analysis of the P13K-AKT-FOXO3a pathway protein expression. (a) The expression levels of the P13K-AKT-FOXO3a pathway proteins were detected with the Western blot analysis. (b) Statistical analysis of the data from the control, 3.2 Gy, 4.0 Gy, and 4.8 Gy groups. Compared with the control and 3.2 Gy groups, ^∗∗^*P* < 0.05.

The expression levels of proteins in the P13K-AKT-FOXO3a pathway were analyzed by the Western blot analysis. As shown in Figure 8, the expression levels of FOXO3a were significantly higher in the 4.0 Gy and 4.8 Gy groups than in the control and 3.2 Gy groups. Moreover, the expression levels of P13K and AKT were significantly lower in the 4.0 Gy and 4.8 Gy groups than in the control and 3.2 Gy groups. There was no significant difference in these protein expression levels between the control and 3.2 Gy groups. These results indicate that radiation can increase the expression levels of FOXO3a and decrease the expression levels of P13K and AKT, in the radiation-induced ovaries.

## 4. Discussion

With the wide clinical application of radiation technology in tumor diagnosis and treatment, the exposure of the patients to the X-rays has been increased. Radiation can directly or indirectly cause multiple organ damages and gene mutations, inducing tumors, causing aging, and shortening life span. Therefore, the protection from the radiation injuries is of great practical significance in protecting the human body, especially for the highly metabolized and rapidly renewing germ cells. Radiation might lead to POI or even POF, the final phase of POI, which is a female endocrine disease, with the FSH ≥40 IU/L [18]. The main clinical manifestations of infertility (due to high gonadotropin and low estrogen) may cause the long-term menopausal symptoms, inducing harm to the women's physical and mental health. It has been shown that the degree and duration of the radiation-induced ovarian damages depend on the location, dosage, and age of the patients at the radiation therapy [19]. Moreover, the radiation could cause direct damages to the DNA of the follicles, leading to the follicular atrophy and the reduced production of follicle-ovarian hormones and premature menopause. The ovary is a gonad organ, very sensitive to the radiation injuries, with the minimum tolerance of 2-3 Gy and the maximum tolerance of 6.25-12.00 Gy. The incidence of ovarian dysfunction has been significantly correlated with the dosage of radiotherapy [20].

The changes have been due to the effects of radiation, so that a large number of free radicals would be produced, resulting in the lipid peroxidation [12]. It has been generally believed that the biological effects of ionizing radiation mainly include the DNA damages and the damages of other intracellular macromolecules. The X-rays can cause oxidative damages to DNA, resulting in the breakage of the DNA single and/or double bonds, as well as the base damages, and affect the proteins, nucleic acids, and complex lipids by producing the ROS and reactive nitrogen species (RNS) [13]. Lu et al. [15] have also demonstrated that the radiation could not only induce the production of ROS but also lead to the inflammatory injuries and the accumulation of inflammatory mediators. At present, in the model of radiation-induced POF, the whole body irradiation has been applied [14, 17], which can lead to the damages of ovarian function. However, the damages to multiple organs are huge, and therefore, there is a great deal of interference in the study of the therapeutic effects. In order to establish the superior model of clinical disease, the dynamic intensity-modulated radiation therapy was used to treat the models, with the whole course of high-dose radiation to the target area. Meanwhile, the radiation dosages to the normal tissues of the target area would be significantly reduced, therefore achieving high accuracy. The POI model established by this method would be more specific for studying the treatment of the ovarian damages caused by the radiation therapy.

FOXO3a is one of the important downstream transcription factors in the P13K/AKT pathway. The phosphorylated PI3K could activate Akt, thus phosphorylating the FOXO3a, which specifically binds to the structural protein 14-3-3 in the nucleus and transfers from the nucleus to the cytoplasm. Through ubiquitination, the loss of its normal transcriptional activity could lead to reduced apoptosis. When Akt is suppressed, most of the dephosphorylated FOXO3a enters the nucleus and thus enhance the apoptosis. Moreover, Foxo3a plays a key role in the protection of mammalian ovarian function by inhibiting the follicle overactivation and the ovarian granulosa cell apoptosis. It has also been shown that Akt and its downstream molecules can also be negatively regulated by FOXO3a, and the endogenous Akt and Foxo3a can form a complex, promoting the cellular apoptosis [21]. The mice without the FOXO3a gene showed significant activation of the primordial follicles, at around 3 weeks after birth, leading to reduced gonadotropin and early infertility. FOXO3A plays an important role in regulating the ovarian oocyte and follicular development, which leads to autophagy and abnormal cell death [22]. Moreover, it has been shown that FOX03a is a potential therapeutic target for POI [23]. Furthermore, E2 has been shown to downregulate the FOXO3a expression in the granulosa cells and activate the downstream regulatory factor Akt [24]. Because of the phosphorylation of Akt to FOXO3a, the FOXO3a expression is significantly reduced, which ultimately inhibits the apoptosis [24]. Therefore, for the POF by radiation therapy, it is important to reduce the sensitivity of ovarian cells to the radiation therapy and reduce the side effects of radiation therapy, without affecting the killing effect of radiation on tumor cells.

In this study, the Wistar rats with more developed gonads were used. According to the radiation tolerance of ovary, the dosages of 3.2 Gy, 4.0 Gy, and 4.8 Gy were selected for the model establishment. The radiation dosage used herein was within the dosage range from a previous study [20], which had also been confirmed in the preexperiments. After irradiation, the serum levels of FSH and LH in the 4.0 Gy and 4.8 Gy groups were detected, which induced the radiation-induced cycle, decreased E2 level, and increased FSH and LH levels, reaching stable levels at 8 weeks after irradiation, in line with previous findings [18]. The ovary weights were measured in the rats. Our results showed that the ovary weights in the 4.0 Gy and 4.8 Gy groups were significantly lower than the control and 3.2 Gy groups. Moreover, the preantral follicles, antral follicles, and atresia follicles were counted by the H&E staining. The antral follicles in the 4.8 Gy and 4.0 Gy groups were significantly less than the control and 3.2 Gy groups. The rats of the control and 4.8 Gy groups were monitored at 6 and 8 weeks after radiotherapy. Our results showed that the ovarian atrophy became smaller, and reduced antral follicles were observed, except for the atresia follicles, in line with previous findings [17]. There was the estrous cycle in the 4.0 Gy group, and preantral follicles could be seen in the ovary. Our results showed that the POI model could not be established after the 3.2 Gy single irradiation, while the ovarian function of the 4.8 Gy group reached the end stage of POF, which is not conducive to the investigation of the therapeutic effects. Moreover, in this study, the levels of IL-6, IL-1*β*, and TNF-*α* were determined, and the oxidative damages were assessed. Our results showed that the levels of IL-6, IL-1*β*, and TNF-*α*, as well as the oxidative damages, in the 4.8 Gy and 4.0 Gy groups were significantly higher than the control and 3.2 Gy groups, in line with previous findings showing that as the dose of radiotherapy increases, the inflammatory damages and oxidative damage of the rat ovarian tissue would be increased [15]. In the study of the P13K-AKT-FOXO3a signal pathway, the expression levels of FOXO3a in the 4.8 Gy and 4.0 Gy groups were significantly higher than the control and 3.2 Gy groups, while the expression levels of P13K-AKT were significantly decreased. Therefore, our results suggest that the radiation-induced ovarian damages may lead to the P13K-AKT downregulation through inflammation and oxidative damages, and most of the dephosphorylated Foxo3a entering the nucleus to accelerate the granulosa cell apoptosis, in line with previous findings showing that the oxidative damages and inflammatory damages activate the P13K-AKT-FOXO3a signaling pathway [22–24]. However, the limited sample size would be the limitation of this study, which needs to be expanded in further in-depth studies in the future.

## 5. Conclusions

In conclusion, the radiation dosage of 4.0 Gy could be used to establish the POI rat models, which met the diagnostic criteria for POI and could not be saved, without causing excessive ovarian failure. Therefore, the ovarian injuries may lead to oxidative damages and inflammatory injuries, downregulate the expression levels of P13k and Akt, and upregulate the expression levels of FOXO3a, thus leading to plenty of follicular atresia in the ovary.

## Data Availability

The data that support the findings of this study are available on request from the corresponding author.
